# The experience of brace treatment in children/adolescents with scoliosis

**DOI:** 10.1186/1748-7161-1-8

**Published:** 2006-05-22

**Authors:** Despina Sapountzi-Krepia, Maria Psychogiou, Darin Peterson, Vassiliki Zafiri, Eugenia Iordanopoulou, Fotini Michailidou, Anastassios Christodoulou

**Affiliations:** 1 Nursing Department, Technological Educational Institute of Thessaloniki, Thessaloniki, Greece; 2 Department of Nursing Science, University of Kuopio, Kuopio, Finland; 3 Anthropological Museum, Medical School, University of Athens, Greece; 4 Medical School, Aristotelian University of Thessaloniki, Greece

## Abstract

**Background:**

Idiopathic scoliosis is a chronic illness with several different braces used for its treatment. Brace treatment during childhood/adolescence can produce stress. There are studies supporting that it can decrease body-image perception while other studies support that it has no such effect.

The purpose of this study was to explore the experience of brace treatment in children/adolescents with scoliosis. The aim was to investigate which feelings are created by the bracing experience in children/adolescents with scoliosis and what are the children/adolescents' with scoliosis opinions of the support provided to them by health-care professionals and by their families.

**Methods:**

We conducted interviews with the help of a semi-structured interview guide in order to address the topic of the experience of brace treatment. A convenient sample of twelve children and adolescents with scoliosis was selected from patients attending follow-up appointments at the Outpatient Scoliosis Clinics of two Greek hospitals. The data was analysed using the method of content analysis.

**Results:**

Patients in the sample were 10–16 years old and they were mainly females (71%). Almost all of the participants reported having to deal with stress, denial, fear, anger, and shame. They were satisfied with the information they received regarding their condition and therapy. However, the information was not accompanied by support from the health care professionals. They reported that they were receiving support mainly from their families, friends, and classmates.

**Conclusion:**

The present study is contributing to the development of a better understanding of significant issues related to the experience of bracing therapy. It is clear that scoliosis children/adolescents have to be provided with support during the long period of bracing. It is apparent that those children/adolescents have unmet needs for care and health professionals and policy makers should try to find a way to address those needs.

## Background

Idiopathic scoliosis is a chronic illness associated with structural deformity of the spine and is characterised by lateral curvature and vertebral rotation of a series of vertebrae from the middle line of the spinal column. The aetiology of scoliosis is still unknown [1]. There are three main types of scoliosis, according to the age in which scoliosis starts; infantile, childhood, and adolescent scoliosis [2]. However, adult scoliosis is also another type of scoliosis, which can be either non-diagnosed scoliosis from an earlier age, or scoliosis that actually started in adulthood [3].

The most common form of scoliosis is adolescent idiopathic scoliosis, which is more common in females. The scoliosis size is determined by the measurement of the angle of the scoliosis curve, which is called the Cobb-angle. The treatment of idiopathic scoliosis includes early detection, exercises, brace treatment, and surgical treatment [4,5].

The history of brace treatment for scoliosis goes back to 1800, and the development of braces has been based on empirical trial and error [3,4]. Around the middle of the 20^th ^century, a step forward was made when the Milwaukee brace was introduced. The Milwaukee brace was followed in the 1970's by the introduction of the Boston brace [6]. Today, a variety of braces are used for scoliosis treatment in many countries including the Milwaukee brace, the Boston brace, the Cheneau brace, the Dynamic Derotation Brace, the Stagnara brace, and the Mitchell brace [7-10]. In Greece several kind of braces are used for scoliosis treatment [10]. Veldhuizen et al. [7] argue that brace design has focused to a great extent on the brace's efficacy, and in this way it has overlooked teenagers' need for good physical appearance. Teenagers do not want to look different from their peers and, although bracing is not associated with pain, it is disturbing for patients. There is evidence that bracing tends to affect them psychologically due to disturbances of their perceptions of self and body image [7,11-18].

Diseases affecting the body structure can alter the body image and reactions to such alterations are influenced by many factors, including family and cultural attitudes [19,20]. Following the scoliosis diagnosis and the start of bracing therapy, there is a need for a certain amount of adjustment to the new situation that produces stress, while uncertainty about the success of the therapy and changes in lifestyle further burdens the patient [18]. Furthermore, several studies found that brace treatment itself tends to produce stress [21-24] and therefore emotional support to braced children and adolescents is required. Such provision is regarded useful by patients as well [17].

Studies of the psychological effects of bracing have been focused on the long-term effects of scoliosis treatment, on non-compliance, and the ability to deal with the stress of chronic disability, while assuming that the patients' psychological well-being is considered quite good [25-27]. However, adolescence is recognised as a sensitive period of life and, when it is compounded with the biopsychosocial effects of scoliosis and of brace-treatment, it can produce immense psychological stress [12,14,15,28,29]. Adolescence requires special adjustment in the presence of a chronic disease [18].

Quality of life is a complicated issue and, as Wood-Dauphinee [30] argues, it is quite new in the field of spine research. It should therefore be better understood and deepened. Quality of life in scoliosis is not only referring to psychological aspects of brace treatment, but includes several other domains such as mental health, physical health, social functioning, and aesthetic domain [31,32].

Regarding the quality of life of children and teenagers with scoliosis, Reichel and Schanz [18] argue that scoliosis can be a risk factor for psychological impairment, particularly in patients undergoing brace-treatment. Furthermore, Climent and Sanchez [33], who studied the impact of the type of brace therapy, found that bracing affects the patient's quality of life. The Milwaukee brace caused a significantly greater impairment in the quality of life of their subjects in comparison to the subjects treated with the Boston brace and TLSO as well as with the Charleston.

Other studies [34-36] also support that bracing impairs the quality of life of patients and most of these implications, although to a lesser extent, seem also to be present in patients using the Boston brace. Sapountzi-Krepia et al. [17] argue that adolescents with scoliosis have a lower body image perception than their non-scoliosis peers and, within the scoliosis group, girls have a lower body-image perception in comparison to boys. It is also supported that a good relationship between mother and child has a positive influence on brace treatment [27], while support from siblings and health care professionals is also encouraged [17,18]. However, there are also contradicting opinions supporting that adolescent body image perception is not lowered by bracing [25-27], while Ugwonali et al. [6] claim that bracing does not decrease quality of life.

Most research on scoliosis consists of quantitative studies, while qualitative studies on this topic are lacking. This stimulated our interest in undertaking this research project in order to study how children and adolescents with scoliosis describe their experience of brace treatment.

The aim of the study was to explore the experience of brace treatment of children and adolescents with scoliosis and to get answers in the following areas.

a) What feelings are created by the bracing experience in children/adolescents with scoliosis?

b) What are the children/adolescents with scoliosis opinions of the support provided to them by their families and by health-care professionals?

## The sample

A convenient sample of twelve children and adolescents with scoliosis was selected from patients attending follow-up appointments at the Outpatient Scoliosis Clinics of two Greek hospitals. The participants had to meet the following inclusion criteria:

1) Age between 10–18 years old.

2) They were undergoing brace treatment for more than 6 months.

3) They were undergoing brace treatment for twelve or more hours per day.

Adolescents with scoliosis fulfilling the above mentioned criteria and their parents were approached by the researchers. They were informed about the purpose of the study, and they were assured that participation was voluntary and confidential, before being asked to participate. Permission for the patients' participation was obtained from their parents.

## Ethics

The Board of the Nursing Department of the Technological Educational Institute of Thessaloniki acting as an ethics committee approved the study. Permission to carry out the study in the hospitals was given by hospital authorities.

## Instrument and data collection

A semi-structured interview guide was used to help address the topic of the experience of brace treatment. Information on demographic and social characteristics of participants was also collected.

Interviews were conducted in a hospital office, and lasted from 45 to 60 minutes, depending on the response time of the patients. All interviews were audio-taped. No additional information was obtained and no contact with the participants occurred after the interviews were completed. The participants did not receive any compensation for their participation in the study.

## Data analysis

**Figure 1:**
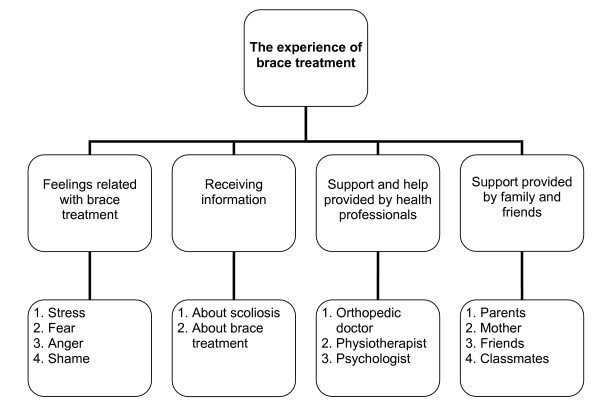
Final categories and subcategories of the content analysis.

The data was analysed using the method of content analysis [37]. All interviews were transcribed. Three members of the research team repeatedly and carefully read the transcribed texts in order to achieve a thorough understanding of the material. After reading the material, the researchers wrote down codes that were freely generated from the material. They made independent lists of classifications, including categories and subcategories according to the codes, then categories and subcategories were divided into groups. Afterwards the three researchers discussed their lists and made any necessary adjustments to form a final list. The final categories and subcategories are illustrated in Figure 1.

The demographic information was analysed separately.

## Results

Patients in the sample were mainly females (71%) and their age ranged from 10–16 years. All participants were primary or secondary school pupils and were living in two major cities of Greece. There are four major theoretical categories related to the children's perceptions of their:

1) Feelings related with brace treatment,

2) Experiences related to receiving information,

3) Experience of support and help provided by health professionals, and

4) Experience of support provided by family and friends.

### Feelings related with brace treatment

Scoliosis adolescents expressed their feelings and memories in relation to the experience of bracing. Almost all (11/12) of the interviewed scoliosis children/adolescents reported having experienced stress, fear, anger, and shame. Examples of the participants' statements about their feelings are listed in Table 1.

**Table 1:** Examples of statements on feelings related with brace treatment

**Denial**
"I didn't want to hear that I have something serious that requires such therapy..."
**Fear**
"Many times I was afraid to look at myself in the mirror..."
"I felt a huge weight on me...I was a little bit afraid about that..."
**Stress**
"I felt as if my breathing was stopping..."
"The first day at school, I felt very ill-at-ease about how my classmates will treat me..."
"I was worried about the brace being visible under my clothes and I didn't want to go outside the house, not even to have fun..."
**Anger**
"At first I was angry with my parents, with the doctor, with myself..."
"During the first days I was very angry and I was behaving hostile towards everybody..."
"My life had changed and I was getting angry about it..."
"I was very angry, I wasn't me, I was someone else, a snail that was carrying its shell..."
**Shame**
"I was ashamed to go out..."
"I was ashamed about wearing the brace at school..."
"I was ashamed when my classmates were looking at me strangely..."

### Experiences related to receiving information

Almost all the subjects (11/12) reported positive experiences related to receiving information about their condition and therapy. They stated that doctors provided them with information related to scoliosis and bracing during follow up visits. Table 2 displays examples of the subjects' responses regarding the information provided.

**Table 2:** Examples of statements in relation to receiving information about scoliosis and its therapy

"The doctor informed me about scoliosis the first time..."
"The doctor talked to me, he told me what scoliosis is and what therapy I had to have..."
"Basically, it was the doctor who I was in contact with and we were talking about my disease..."
"Every time that I was going to the doctor, he was talking to me about my condition..."
"I don't think that the doctor really wanted to deal with me..."

### The experience of support and help provided by health professionals

This category examines the support and help that the children/adolescents received from the health professionals during their hospital visits. All the children/adolescents stated that they had the chance to discuss with their doctor and some of them (3/12) with a physiotherapist about their physical problems. However, all of them stressed that this was not enough. In relation to their problems, two of the participants stated that they had not openly expressed their feelings in front of the doctor. Only one of the participants had visited a psychologist. None of the participants mentioned the nurses as members of the health professionals' team, nor for offering any help to them. Examples of the participants' statements about the support provided to scoliosis children/adolescents by health professionals are listed in Table 3.

**Table 3:** Examples of statements about the support provided by health professionals

"The doctor and the physiotherapist explained to me how to sleep wearing the brace, and that was all..."
"The psychologist said to me that I should not be ashamed of my appearance..."
"The doctor encouraged me to change my daily habits, but that was only in the follow up visits..."
"The doctor was talking to me and was making me feel more comfortable..."
"Whenever I knew that I would go to the doctor, I was a little bit afraid about what he was going to say about my condition..."
"I think that apart from the doctor no-one else dealt with me and my problems..."

### The experience of support provided by family and friends

Almost all the participants (11/12) reported that they have received support mainly from their families, friends, and classmates. However, one girl reported a difficult experience as she received critical comments that she was not accepting the reality of her condition. Excerpts from the subjects' answers related to support received from family and friends are presented in Table 4.

**Table 4:** Examples of statements about the support provided by family and friends

"My parents, and mostly my mother, were constantly by my side..."
"My mom was helping me always in everything and was making me feel good about myself..."
"My friends and classmates encouraged me a lot..."
"I was feeling that only my parents could understand me..."
"Regardless of the support provided, when I was alone I was thinking about it, it concerned me..."
"One girl-classmate was criticizing me for not accepting my condition... I believed that she was considering me handicapped, that she didn't think that I will get well, and that bothered me a lot..."

## Discussion

There are several methodological concerns that should be discussed before the interpretation of the study findings. The age range (10–16) of our sample and the time wearing of the brace (twelve or more hours per day) were set because in this first qualitative study on scoliosis bracing in Greece we wanted to capture the general idea about the problems faced by the population of braced children. Afterwards, it may be possible to proceed with further research according to age groups and full- or part-time brace wearing.

This first qualitative study on scoliosis bracing in Greece is contributing to the development of a better understanding of significant issues related to the experience of brace treatment for scoliosis children/adolescents. Furthermore, our findings may be of interest to doctors, physiotherapists, occupational therapists, nurses, and other health and welfare professionals interested in the topic. The findings could be used for improving the patients' compliance on brace therapy. Moreover, our findings may be of interest to health planners, people involved in public health policy and health authorities. They can also have an influence on the formulation of specific policies that will include patient support during bracing therapy.

Our results support the arguments of various authors that bracing is a disturbing experience for patients and has an influence on their psychology [7,12-18] since the participants reported that during brace therapy they had to deal with stress, denial, fear, anger, and shame.

The vast majority of the subjects were satisfied with the information provided by doctors regarding illness and therapy. However, the lack of support from specialised professionals when the "bad" news of the necessity of brace-treatment is conveyed to children, as well as the provision of minimal emotional support, mainly by orthopaedic doctors during follow up visits, is apparent in the answers given by the subjects. The data obtained from the qualitative interviews support the claims of Sapountzi-Krepia et al. [27] that there is a gap in the provision of emotional support to adolescents with scoliosis who receive brace treatment in Greece.

It is important to note that almost all the participants feel that they receive support from their families and some times from their friends as well. The parents, and especially the mothers, seem to be very supportive and they help the subjects feel good about themselves. This finding is in concurrence with the argument of Olafsson et al. [27] that a good relationship between mother and child has a positive influence on brace treatment. Some of the participants also mentioned that their friends were supportive and encouraging, while one mentioned that a girl-classmate was criticizing her for not accepting her condition. Support from family and friends is essential for any patient, especially for children and adolescent patients, but it should be accompanied by support from health professionals.

## Conclusion

Considering these findings, it must be taken into account that scoliosis is a chronic illness starting mainly in late childhood. Therefore, scoliosis patients enter adolescence with a chronic illness and they find their body imprisoned in a plastic brace. Adolescence is recognized as a difficult psychological period in its own right and when it is compounded by the biopsychosocial effects of scoliosis and the brace treatment it can produce stress [12,14-16,21-24,28,29]. Thus, scoliosis children/adolescents have to be provided with support during the long period of bracing.

Furthermore, as it becomes clear from the results, scoliosis children/adolescents have unmet needs for care. Orthopaedic doctors, other health professional, and policy makers should take a closer look at the needs of those clients of the Greek National Health System and try to find a way to address those needs. The introduction of new policies for scoliosis patients and their parents as earlier argued by Sapountzi-Krepia et al. [17] may include the provision of health education and advice about scoliosis and bracing from a team of nurses or health visitors, as an important first step. In addition, the provision of support by psychologists to selected cases is also considered appropriate.
